# Factors Associated With Medical and Nursing Students’ Willingness to Donate Organs

**DOI:** 10.1097/MD.0000000000003178

**Published:** 2016-03-25

**Authors:** Makmor Tumin, Khaled Tafran, Li Yoong Tang, Mei Chan Chong, Noor Ismawati Mohd Jaafar, NurulHuda Mohd Satar, Nurhidayah Abdullah

**Affiliations:** From the Department of Administrative Studies and Politics (MT), Universiti Malaya; Institute of Research Management and Monitoring (KT), Universiti Malaya; Department of Nursing (TLY, CMC), Universiti Malaya; Department of Applied Statistics (NIMJ), Universiti Malaya; Department of Economics (NMS, NA), Universiti Malaya, Kuala Lumpur, Malaysia.

## Abstract

Malaysia suffers from a chronic shortage of human organs for transplantation. Medical and nursing students (MaNS) are future health professionals and thus their attitude toward organ donation is vital for driving national donation rates. This study investigates MaNS’ willingness to donate organs upon death and the factors influencing their willingness. A cross-sectional design was used with a sample of 500 students (264 medical and 236 nursing) at the University of Malaya. A self-administrated questionnaire was used. The responses were analyzed by using descriptive statistics and multiple logistic regression. Of all respondents, 278 (55.6%) were willing to donate organs upon death, while the remaining 222 (44.4%) were unwilling to donate. Only 44 (8.8%) had donor cards. The multiple logistic regression revealed that the minorities ethnic group was more willing to donate organs than Malay respondents (adjusted odds ratio [aOR] = 1.98, *P* = 0.010). In addition, medical students were more willing to donate than nursing students (aOR = 2.53, *P* = 0.000). Respondents who have a family member with a donor card were more willing to donate than respondents who do not (aOR = 3.48, *P* = 0.006). MaNS who believed that their religion permits deceased donation were more willing to donate than their counterparts (aOR = 4.96, *P* = 0.000). Household income and sex were not significant predictors of MaNS’ willingness to donate organs upon death. MaNS have moderate willingness, but low commitment toward deceased organ donation. Strategies for improving MaNS’ attitude should better educate them on organ donation, targeting the most the Malay and nursing students, and should consider the influence of family attitude and religious permissibility on MaNS’ willingness.

## INTRODUCTION

Malaysia has a tragic gap between demand for and supply of human organs. In December 2013, >18,000 patients were registered on the waiting list for a kidney transplant,^1^ but only 94 kidney transplants were performed in that year.^2^ The shortfall in human organs is chiefly attributed to the low deceased donation rate. In 2013, Malaysia reported a very low deceased donation rate of 0.5 donations per million population (PMP), which was far lower than the PMP deceased donations achieved in Spain (35.1), USA (26.0), UK (20.8), Brazil (13.2), South Korea (8.4), and Chile (6.4) in that year.^3^

The attitude of healthcare professionals toward organ donation is deemed as one of the most important factors that influences donation rates. Health professionals are responsible for identifying potential donors, contacting organ donation coordinators, and approaching the families of potential donors to gain their consent. Moreover, they can also serve as a role model and promote organ donation to the public.^4–6^ Medical and nursing students (MaNS) are future health personnel and thus their attitude toward organ donation is also of huge importance.^6–9^

Studies in Malaysia show that many factors influence people's willingness to donate organs upon death, such as ethnicity,^5,10,11^ income,^10^ sex,^5,10^ and religion.^5,10^ Family attitude has also been found to affect deceased organ donation.^11^

To our knowledge, no studies have thus far explored healthcare students’ attitude toward organ donation in Malaysia. To bridge this information gap, this study sought undergraduate MaNS’ attitude on deceased organ donation to assess their willingness to donate organs upon death and examine the associated driving factors.

## METHODS

To achieve the study's objectives, on the light of previous studies on organ donation,^5,12–16^ experts in social science and medicine (Makmor Tumin, Li Yoong Tang, and Mei Chan Chong) collaborated to produce a self-administrated questionnaire. The questionnaire was prepared and distributed in English.

The questionnaire was pilot tested and revised and the final version was printed and distributed to undergraduate students at the Faculty of Medicine and Universiti Malaya Medical Centre, Kuala Lumpur, Malaysia. Two trained enumerators were asked to approach students between classes in the library and student corner at the faculty from October to December 2014.

The pilot test was performed 1 month before the final questioners were distributed, where a group of medical and nursing students were invited to read and answer the questions of the initial questionnaire. Few changes were done after the pilot test. The changes included, adding a short description about the importance of organ donation to save the life of others and to improve the national health in general, clarifying that the organ donation after death (not living donation) is what we are asking about in the questionnaire, and giving categorical options on household income instead of asking respondents to give specific figures as it was in the initial questionnaire.

In 2014, there were 933 medical and 600 nursing students registered at the University of Malaya. Based on earlier research in Malaysia,^5,11,12^ we expected the probability of MaNS’ willingness to donate organs to be about 40%. Therefore, for a confidence level of 95% and a precision level of 5%, a minimum of 384 respondents were needed to meet the study objectives.^17^ Although the larger the sample size is preferable, the time and money needed for data collection is a concern for all researchers.^17^ Thus, using the available resources, we distributed 600 questionnaires (300 to nursing students and 300 to medical students) and collected 500 completed questionnaires (264 from medical students and 236 from nursing students), resulting in a response rate of 83.3%. Our enumerators were trained to briefly describe the content of the questionnaire and to ask students to complete the questionnaire while the enumerator is attendant. The enumerator reported that the majority of students who did not complete the questionnaire apologized for not doing so stating that they are busy or not having enough time. Some students, however, did not give any justifications for not participating in the survey.

Respondents were first asked to fill in their demographic and socioeconomic information. Next, they were presented with the following question: “are you willing to donate your organs upon death?” Then, respondents were asked whether they believe that their religion permits deceased organ donation or not. Lastly, they were asked if they have a family member who has a donor card.

Statistical analyses were performed by using SPSS 20.0 (SPSS Inc, Chicago, IL). Logistic regression was used to estimate the factors associated with willingness to donate organs upon death. The dependent dummy variable was given the value “1” for willingness and “0” otherwise. The independent variables had 3 themes capturing the influence of demographics, family, and religious permissibility on willingness to donate organs (dependent variable).

First, the association between the dependent and independent variables was tested by using bivariate analyses (Pearson chi-square test). Second, the factors that showed a significant association with the dependent variable were regressed together against the dependent variable in multiple logistic regression to obtain adjusted odds ratios (aORs). The 5% significance level was used as the criterion. To avoid overfitting the estimates, we assured a minimum of 10 outcome events per predictor variable.^18,19^

All human studies were reviewed by the University of Malaya Research Ethics Committee (Reference Number: UM.TNC2/RC/H&E/UMREC-35).

## RESULTS

Table 1 reports the breakdown of respondents and their willingness to donate organs upon death. The sample comprised 52.8% medical students and 47.2% nursing students. Almost three-quarters were females (72.4%). The sample ethnic breakdown resembled the Malaysian ethnic profile, which consists of Malay/indigenous (61.9%), Chinese (22.6%), Indian (6.7%), and others (0.7%).^20^ Our sample breakdown was as follows: 66.6% Malay, 27% Chinese, and 5.6% Indian, and others. Because the “Indian and others” category was relatively small (28 respondents), all non-Malay respondents were grouped into 1 category named “Minorities.” Of the 500 respondents, 278 (55.6%) stated that they were willing to donate organs upon death (Table 1). Of these respondents, 44 (8.8%) were donor card holders (35 medical students and 9 nursing students).

**TABLE 1:**
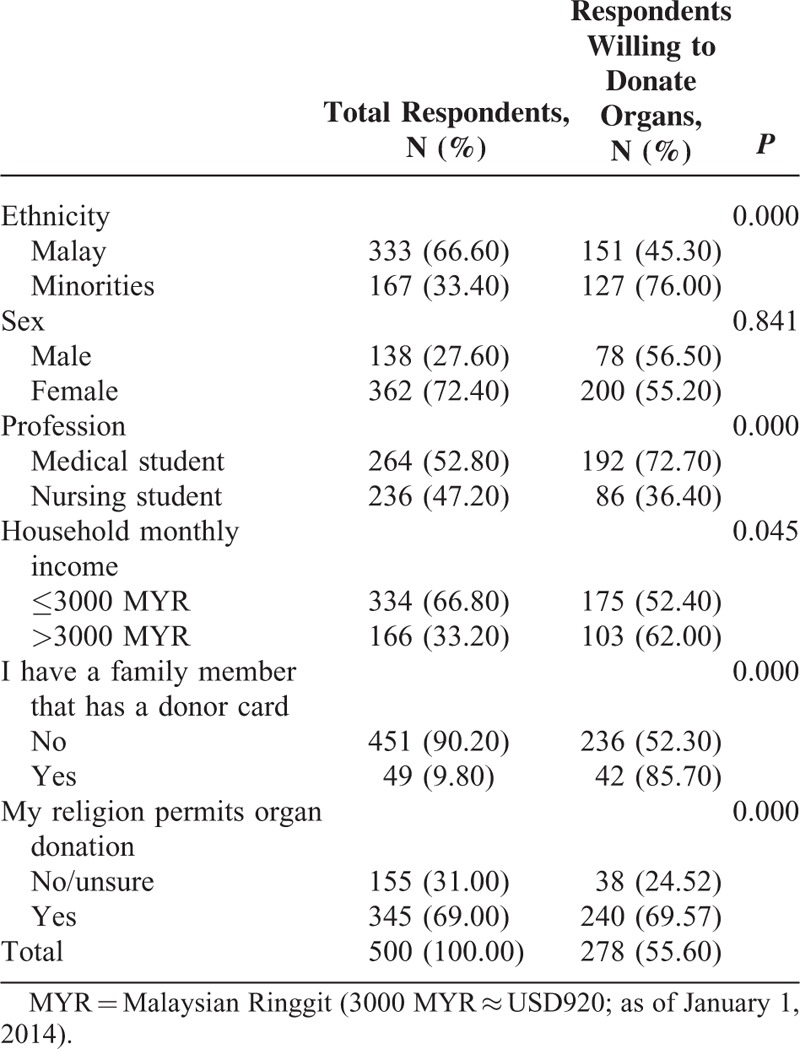
Bivariate Analysis of Respondents’ Willingness to Donate Organs Upon Death

The bivariate analyses (Table 1) revealed that ethnicity, profession, household income, having a family member with a donor card, and believing that religion permits organ donation were significantly associated with willingness to donate organs upon death (*P* < 0.05). On the other hand, sex was not associated with willingness to donate.

The results of the logistic regressions presented in Table 2 show that the minorities ethnic group was twice as willing to donate organs as Malay respondents (aOR = 1.98, *P* = 0.010). Medical students were 2.5 times more willing to donate than nursing students (aOR = 2.53, *P* = 0.000). Respondents having a family member who has a donor card were 3.5 times more willing to donate than their counterparts (aOR = 3.48, *P* = 0.006). Respondents who confirmed that their religion permits organ donation were about 5 times more willing to donate than their counterparts (aOR = 4.96, *P* = 0.000). Household monthly income was an insignificant predictor of willingness in the multiple logistic regression.

**TABLE 2:**
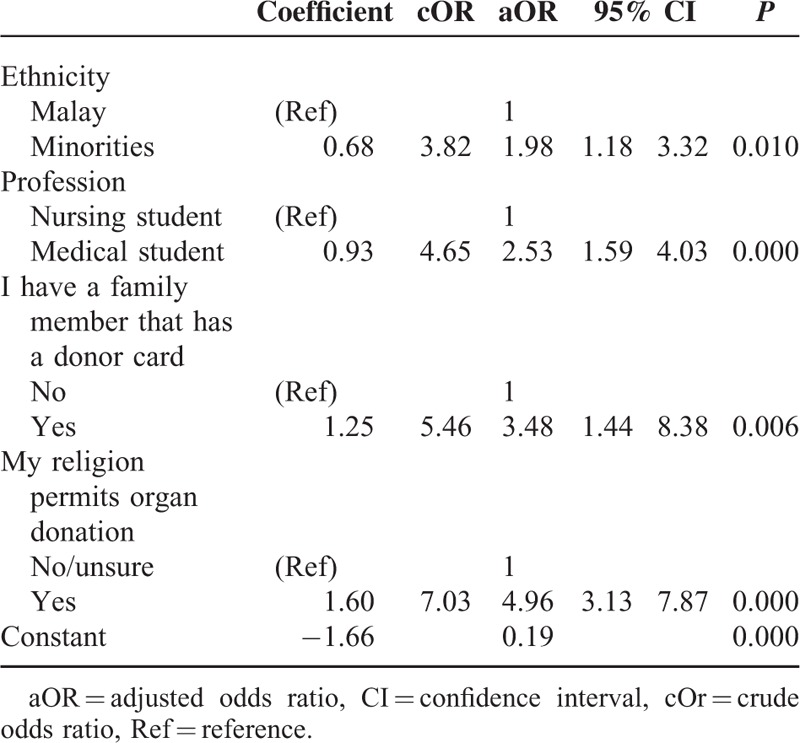
Factors Associated With MaNS’ Willingness to Donate Organs Upon Death, Multiple Logistic Regression

The variance inflation factor value was <1.2 across all the independent variables included in the multiple logistic regression, indicating no collinearity problem in the model. The *P* value of Hosmer and Lemeshow test (*P* = 0.264) indicates that our model fits the data well.

## DISCUSSION

Although MaNS’ contribution to the deceased donations pool as donors is important, their role (as future health professional) in facilitating organ donation process is of greater significance in increasing deceased donations rates.^6–9^ With 55.6% of MaNS willing to donate organs, it could be said that they have a moderate attitude toward deceased organ donation. However, MaNS’ willingness is higher than that of the Malaysian public (44%),^11,12^ and of Malaysian health professionals (47.8%).^5^ On the other side, the fact that only 44 (8.8%) of total respondents were donor card holders shows a low level of commitment toward organ donation among MaNS, especially compared with the level of commitment among healthcare students in the United Kingdom (43%–74%),^7^ Germany (25%–43%),^6^ and the United States (61%).^21^ The critical importance of MaNS (as future health professionals) in organ donation and transplantation^6–9^ urges intervention policies to enhance their attitude and behavior toward organ donation.

Our results revealed that ethnicity is a significant predictor of MaNS’ willingness to donate organs upon death. This accords with previous research on Malaysian healthcare professionals^5^ and the Malaysian public.^10,11^ Similarly, ethnicity was found to be significantly associated with willingness in the United States.^22,23^ These findings imply that strategies for improving MaNS’ willingness to donate organs should be ethnic-specific and should target the Malay ethnic group most heavily. These strategies should not only focus on increasing awareness and willingness to donate organs as suggested in pervious research,^5,7^ but also on encouraging MaNS to obtain donor cards.

Islam (61.3%), Buddhism (19.8%), Christianity (9.2%), and Hinduism (6.3%) are prominent religions in Malaysia,^20^ and all permit organ donation.^24,25^ Despite this fact, 31% of MaNS were unaware about the religious permissibility of organ donation. Our results thus provide evidence that MaNS’ willingness is highly influenced by their beliefs about the religious permissibility of organ donation.

Having a future health professional who believes that organ donation is against his or her religion would leave direct and indirect negative implications on deceased donations rates. The direct effect is that such a health professional would not donate his or her organs after death. The indirect effect, which could be much more acute, is related to the potential moral objection from such a professional to facilitate a clinical practice that contradicts his or her religious belief.^26^ In other words, almost all religions consider a person helping others to commit a sin as sinful. Therefore, it is likely that a health professional would hinder or at least would not facilitate others’ donations in case he or she believes that it is against his or her religion. These emphasize that signifying the religious permissibility of organ donation to MaNS is key to improve deceased donations in Malaysia.

The results of this study revealed that medical students have greater willingness to donate than nursing students. This accords with the findings of a study of MaNS in Greece,^27^ but opposes the outcomes of another study in England.^7^

Earlier research revealed that family members influence an individual's decision to donate organs.^11,28^ This accords with our findings, which showed the huge influence of family attitude on MaNS’ willingness to donate organs. This fact suggests that MaNS’ willingness could be improved by enhancing their families’ attitude toward organ donation.

Our logistic regressions showed that sex and household income were not significant predictors of MaNS’ willingness to donate, which is in line with the studies of Chinese medical students.^29,30^ However, a study of Malaysian health professionals^5^ and another of the Malaysian public^10^ both found sex to be a significant predictor of willingness to donate organs.

Our earlier study explored the possibility of shifting from the informed consent system to the presumed consent system as a strategy to improve deceased donations in Malaysia.^16^ The study concluded that Malaysia is not ready yet for such a shift owing to the large public objection to the new system, low family consent rates on relative's donations, and the absence of adequate medical infrastructure.^16^ Nevertheless, the implementation of the presumed consent system may increase deceased donations if the latter mentioned barriers were eliminated. The default organ donation system can be supported or hindered by health professionals’ attitude toward organ donation both directly by their own donations and indirectly as they are the key in facilitating others’ donations.^6–9^ Hence, any effort to change the organ donation system in Malaysia should take MaNS’ perspective in consideration. Consequently, future research should focus on MaNS’ attitude toward the presumed consent system.

## LIMITATIONS

Similar to other studies,^6,7^ this study used observation from a single public university and a nonprobability sampling; thus, the views of MaNS in other institutions may vary. However, we believe that our results provide a very important insight into its context, as the University of Malaya is the leading educational institution in Malaysia. In addition, the correlation between variables does not necessarily mean causation. Future studies may seek time-variant observations to establish causal models.

## CONCLUSIONS

MaNS’ attitude and commitment toward organ donation should be improved to increase organ donation among future healthcare professionals which would, in turn, indirectly improve deceased donations among the public. A study from the US^21^ and another from Germany^31^ showed that by exposing MaNS to educational programs on organ donation, their attitude could be significantly improved. In the light of our results, we suggest that educational programs for enhancing MaNS’ willingness to donate organs are needed. These programs should focus on Malay and nursing students as well as approach MaNS’ families. The crucial message of these educational programs should be the religious permissibility of deceased organ donation.
